# Draft genome sequence of multi-resistant *Salmonella enterica* subsp. *enterica* serovar Rissen strain 19CS0416 isolated from Vietnam reveals *mcr-1* plasmid mediated resistance to colistin already in 2013

**DOI:** 10.7150/jgen.42790

**Published:** 2020-07-03

**Authors:** Belen Gonzalez-Santamarina, Anne Busch, Silvia Garcia-Soto, Mostafa Y. Abdel-Glil, Jörg Linde, Reinhard Fries, Diana Meemken, Helmut Hotzel, Herbert Tomaso

**Affiliations:** 1 Friedrich-Loeffler-Institut, Institute of Bacterial Infections and Zoonoses (IBIZ), Naumburger Str. 96a, 07743 Jena, Germany.; 2 Institute of Food Safety and Food Hygiene, Section Meat Hygiene, Freie Universität Berlin, Königsweg 67, 14163 Berlin, Germany.; 3 Department of Anesthesiology and Intensive Care Medicine, University Hospital Jena, Am Klinikum 1, 07747 Jena, Germany.

**Keywords:** *Salmonella* Rissen, multidrug resistance, colistin, genome sequence, pig.

## Abstract

We report the first draft genome sequence of a *Salmonella* strain with plasmid-mediated resistance to colistin encoded by *mcr-1* gene in Vietnam. *Salmonella enterica subsp. enterica* serovar Rissen was isolated from a Vietnamese pig slaughterhouse in 2013. We can confirm that *mcr-1* gene is identical to the first reported *mcr-1* gene of the *Escherichia coli* strain SHP45, isolated in 2015 from a Chinese pig. The plasmid containing this gene in the strain 19CS0416 was highly related (96.86% identity) to the plasmid (pHNSHP45) contained in this Chinese strain. Moreover, this plasmid was determined to be 100% identical to a plasmid (p13P477T-7) belonging to an *Escherichia coli* (13P477T) found in Hong Kong during the same year in pigs. Our results will aid in understanding the dissemination of *mcr-1* gene in East Asia, dating back to as early as 2013.

## Text

Salmonellosis, a foodborne disease caused by *Salmonell*a spp., is of global concern due to the high number of infections and increasing resistance of the bacteria to antibiotics. This zoonotic pathogen occurs in many food-producing animals and can be transmitted by raw meat, eggs, and dairy products 1. Non-typhoidal *Salmonella* are a major cause of food-borne gastroenteritis worldwide 2*.* These strains can be host-restricted or host generalists, infecting a broad range of vertebrates 3. Salmonella enterica subsp. enterica serovar Rissen strain is ubiquitous and the most common serovar in pig and human patients with diarrhea in Vietnam 4. We analyzed a *Salmonella enterica subsp. enterica* serovar Rissen that was isolated from a pig carcass in 2013 in a Vietnamese slaughterhouse. We determined the antibiotic susceptibility of isolate 19CS0416 and used next-generation sequencing data to analyze genes conferring antibiotic resistance, virulence genes, and to analize the plasmids.

Bacterial cultivation, species identification, and serotyping were performed using standard procedures according ISO 6579-1:2017 5. Antimicrobial susceptibility of the strain was assessed by determining the clinical minimum inhibitory concentration (MIC) breakpoints in accordance with the European Committee on Antimicrobial Susceptibility Testing 6 using VITEK 2 Compact system (bioMérieux, Marcy-l'Étoile, France). VITEK cards AST-N195 and AST-N248 were employed to determine MIC values (mg/l). The strain was categorized as resistant to ampicillin (MIC of ≥32 mg/l), gentamicin (MIC of ≥16 mg/l), tobramycin (MIC of =8 mg/l) and colistin (MIC of ≥16 mg/l). The strain was categorized as susceptible (increased exposure) (new EUCAST nomenclature) 7 or intermediate (old definition) for: amoxicillin with clavulanic acid (MIC of ≥8 mg/l) and tigecycline (MIC of =2 mg/l). The strain was categorized as susceptible (standard dosing regimen) to all other tested antibiotics piperacillin (MIC of ≤4 mg/l), piperacillin-tazobactam (MIC of ≤4 mg/l), cefalexin (MIC of ≤16 mg/l), cefuroxime-axetil (MIC of ≤8 mg/l), cefotaxime (MIC of ≤1 mg/l), ceftazidime (MIC of ≤1 mg/l), cefepime (MIC of ≤1 mg/l), aztreonam (MIC of ≤1 mg/l), ertapenem (MIC of ≤0.5 mg/l), imipenem (MIC of ≤0.25 mg/l), meropenem (MIC of ≤0.25 mg/l), amikacin (MIC of ≤2 mg/l), ciprofloxacin (MIC of ≤0.25 mg/l), moxifloxacin (MIC of ≤0.25 mg/l), fosfomycin (MIC of ≤16 mg/l), trimethoprim (MIC of ≤0.5 mg/l) and trimethoprim-sulfamethoxazole (MIC of ≤1 mg/l).

For whole-genome sequencing, the strain was grown overnight at 37°C in 3 ml of Luria-Bertani broth (Mast Diagnostica GmbH, Reinfeld, Germany). DNA sequencing libraries were constructed using the Nextera XT Preparation Kit (Illumina Inc., San Diego, CA, USA) in accordance with the manufacturer´s instructions. Paired-end sequencing was performed on the Illumina MiSeq platform (Illumina Inc., San Diego, CA, USA) using a 300-cycle MiSeq reagent kit. In total, 2,351,952 reads were generated. A quality check was performed with FastQC v0.11.5 (available at http://www.bioinformatics.babraham.ac.uk/projects/fastqc/). Reads were *de novo* assembled using SPAdes v3.12.0 in Bayes Hammer mode (--careful) 8 and evaluated with QUAST v4.3 9 in standard settings. The filtering of the sample was performed by removing contigs with coverage less than 5× and size below 500 bases. The filtered genome assembly was represented by 65 contigs with an N50 contig length of 554,575 bp in which the largest contig had 810,994 bp. The average coverage was 116-fold. The combined length of the contigs was 5,083,583 bp with a GC content of 51.92 %. Annotation was performed with RAST Server (v2.0) 10. Annotation features include 5.203 coding sequences (CDS), 10 rRNAs, 79 tRNAs, and 1 transfer-messenger RNA (tmRNA).

Data were submitted and analyzed using an in-house Linux-based bioinformatics pipeline WGSBAC (available at https://gitlab.com/FLI_Bioinfo/WGSBAC). Genes coding for resistance were detected using abricate v08.13 (available at https://github.com/tseemann/abricate). Genotyping was performed with MLST (v2.16.1) showing a sequence type 469 (*aro*C (92), *dna*N (107), *hem*D (79), *his*D (156), *pur*E (64), *suc*A (151) and *thr*A (87)) and the *in silico* serotyping detection was performed with SISTR (v1. 0.2) 11 confirming the serovar Rissen with antigenic formula 6,7,14:f,g:-. Data were analyzed against the databases ResFinder 12 and CARD 13. The databases predicted antimicrobial resistance to penicillins and first-generation cephalosphorins, aminoglycosides, fluoroquinolones, colistin, and lincosamides (*bla*_TEM-1b_*, aac(6′)-Iaa, aac(3)-IId, ant(3”)-Ia, mdf*(A),* floR, tet*(A)*, tet*(M)*, mcr-1, lnu*(F)) (Table S1). Using abricate against the PlasmidFinder 14 database, genomic regions (replicons) of five independent plasmids could be identified (IncI2_1_Delta, IncFIC (FII)_1, ColRNAI_1, Col156_1, Col (BS512)) (Table S2). Plasmid preparation was performed with the QIAGEN® Plasmid Mini Kit (QIAGEN GmbH, Hilden, Germany) according to the manufacturer's instructions. We could confirm the presence of three plasmids in agarose gel electrophoresis 15, 16. One hundred and thirty-seven virulence genes (Table S3) were predicted using abricate and the Virulence Factors Database 17. *Salmonella* pathogenicity islands (SPIs) are suspected to be acquired by horizontal gene transfer and to have an effect on the structure of the genome 18, 19. SPIFinder 20 confirmed three *Salmonella* pathogenicity islands (SPI-2, SPI-8 and C63PI) (Table S4). This is below the average (1-14 SPIs) and may be associated with individual serovar Rissen isolates as described before 20. Therefore, using the virulence genes output predicted by the Virulence Factors Database, we have looked for the virulence genes associated with another SPI 21. Genes related with SPl-I, SPI-3 and SPI-5 were found (Table S4) but not SPI-4. To confirm the presence or absence of SPI-4 the specific pathogenicity island SPI-4 genomic sequence of *S*. *Typhimurium* with accession number KP234070 was downloaded and mapped against our sequence 19CS0416 using Geneious prime (version 11.1.5) (Biomatters, Ltd., Auckland, 1010, New Zealand). The six genes *siiABCDEF* 22 present in this region were found in two different contigs of the studied sequence (Table S4). We could confirm that 7 SPIs were present in this strain (SPI-1, SPI-2, SPI-3, SPI-4, SPI-5, SPI-8 and C63PI).

For the evaluation of the *mcr-1* gene, the program Geneious was used to find this gene in the annotated contigs. It was found in contig number 18. The *mcr-1* gene (annotated as Lipid A phosphoethanolamine transferase by RAST server) was extracted and aligned against the *mcr-1* gene of the *Escherichia* (*E.*)* coli* strain SHP45 plasmid pHNSHP45 published by Liu et al. in 2016. The alignment showed 100% identity with published sequence 23. In the same contig, a replication initiation protein and genes related to Inc1 plasmid were found (Table S5). The sequence has 96.82% identity with the *E. coli* strain SHP45 plasmid pHNSHP45 (accession number KP347127.1). Using BLASTn 24 (https://blast.ncbi.nlm.nih.gov/Blast.cgi) it has been found that our strain has a 100% identity with the *E. coli* strain 13P477T plasmid p13P477T-7 (accession number NZ_CP021103.1), which belongs to a strain published by Ho et al. in 2017 taken from pig feces in Hong Kong in 2013.

Recombination and rearrangement events are driving factors for evolution, and the varying content of plasmids, AMR genes, and SPIs describes the diversity within a species. In further studies, the *mcr-1* gene-containing genomes will be studied for their genomic evolution in a cross-species approach to elucidate the recombination and rearrangement events on the aforementioned genomic features.

**Figure 1:**
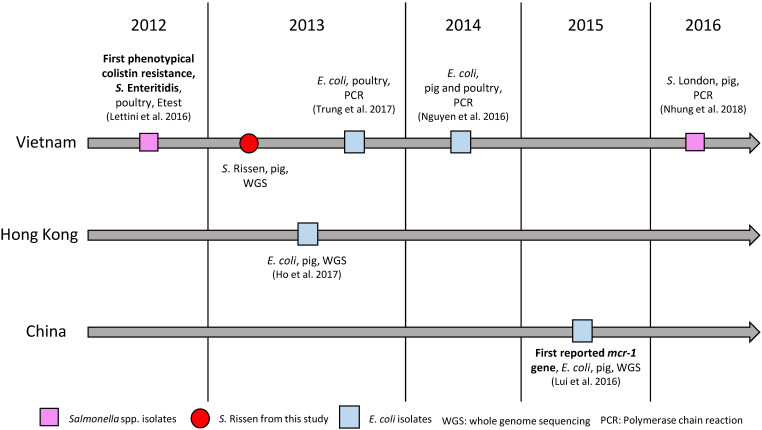
Colistin resistance and *mcr-1* gene in Vietnam, Hong Kong and China.

The emergence of plasmid-mediated colistin resistance gene *mcr-1* was first reported in 2015 in an *E. coli* strain isolated in a pig in China 23. Since then, the gene was found in more than 30 countries 25. In retrospect, the *mcr-1* gene was also reported in *E. coli* isolates from China in the 1980ies 26, as well as in *Salmonella* isolates collected in Germany in 2008 27. In Vietnam, the first detected *mcr-1* gene reported in *E. coli* was detected by PCR in 2013 in poultry 28 and a year later in pigs 29 (Fig. 1). In *Salmonella* spp., the first phenotypically colistin-resistant isolate was found in 2012 in pigs 30, and the first *mcr-1* gene detection in this genus was confirmed by PCR in an isolate from pork between 2016 and 2017 (Fig. 1). Here we report the first draft genome sequence of a *Salmonella enterica* subsp. enterica serovar Rissen strain with a plasmid-mediated resistance to colistin encoded by the *mcr-1* gene from Vietnam, which is identical to the first reported colistin-mediated *mcr-1* gene. The plasmid was closely related (96.86% identity) to the plasmid containing *mcr-1* (pHNSHP45) in the Chinese strain and it showed 100% identity to plasmid p13P477T-7 belonging to an *E. coli* (13P477T) found in Hong Kong in the same year in pigs. Our results will aid in understanding the dissemination of this gene and plasmid in East Asia, dating back to as early as 2013 and in different species as compared to the first reported *mcr-1* carrying strain. The results show the importance of the One Health Approach for the *surveillance* of *resistance* in bacteria in food-producing animals in the food chain, food products, humans and the environment.

### Nucleotide sequence accession number

Whole-genome sequence of multi-resistant Salmonella enterica subsp. enterica serovar Rissen strain 19CS0416 was submitted to SRA database with the accession number: PRJNA591084, BioProject PRJNA591084 (https://www.ncbi.nlm.nih.gov/bioproject/PRJNA591084), and BioSample SAMN13381784 (https://www.ncbi.nlm.nih.gov/biosample/13381784).

## Supplementary Material

Supplementary figures and tables.
