# Supplementary material for: Draft genome sequence of multi-resistant Salmonella enterica subsp. enterica serovar Rissen strain 19CS0416 isolated from Vietnam reveals mcr-1 plasmid mediated resistance to colistin already in 2013
Source: J Genomics. 2020 Jul 3;8:76–9. doi: 10.7150/jgen.42790 (PMC7425045; doi:10.7150/jgen.42790)
Supplement: Supplementary file 1 — Supplementary figures and tables. [file jgenv08p0076s1.pdf]

Draft genome sequence of multi-resistant Salmonella enterica subsp. enterica serovar Rissen strain 19CS0416 isolated from Vietnam reveals mcr-1 plasmid mediated resistance to colistin already in 2013

Belen Gonzalez-Santamarina[1], Anne Busch[1, 3], Silvia Garcia-Soto[1], Mostafa Y. Abdel-Glil[1], Jorg Linde[1], Reinhard Fries[2], Diana Meemken[2], Helmut Hotzel[1] and Herbert Tomaso[1].

[1]Friedrich-Loeffler-Institut, Institute of Bacterial Infections and Zoonoses (IBIZ), Naumburger Str. 96a, 07743 Jena, Germany

[2]Institute of Food Safety and Food Hygiene, Section Meat Hygiene, Freie Universitaet Berlin, Königsweg 67, House 21/22, 14163 Berlin, Germany

[3]Department of Anesthesiology and Intensive Care Medicine, University Hospital Jena, Am Klinikum 1, 07747 Jena, Germany

Supplementary Material

**Antibiotic resistance genes[1]**											
**β-lactamase**		**Aminoglycoside**		**Fluoroquinolones**		**Tetracyclines**		**Colistin**		**Lincosamides**	
**Gene name**	**Accession number**	**Gene name**	**Accession number**	**Gene name**	**Accession number**	**Gene name**	**Accession number**	**Gene name**	**Accession number**	**Gene name**	**Accession number**
bla_{TEM-1b}	AY458016	*aac(6')-Iaa*	NC_003197	*mdf(A)*	Y08743	*tet(A)*	AF534183	*mcr-1*	KP347127	*lnu(F)*	EU118119
		aac(3)-IId	EU022314	*floR*	AF118107	*tet(M)*	X04388				
		ant(3")-Ia	X02340								

Table S1: Antibiotic resistant genes found in the strain *Salmonella enterica* subsp. *enterica* serovar Rissen 19CS0416.

[1] Antibiotic resistance genes were detected using ResFinder and CARD.

**Plasmid[2]**	
**Replicon name**	**Accession number**
IncI2_1_Delta	AP002527
IncFIC(FII)_1	AP001918
ColRNAI_1	DQ298019
Col156_1	NC_009781
Col(BS512)	NC_010656

Table S2: Plasmid replicons found in the strain *Salmonella enterica* subsp. *enterica* serovar Rissen 19CS0416.

[2] Plasmid replicons were detected using PlasmidFinder.

Virulence genes[3]					
**Gene name**	**Accession number**	**Gene name**	**Accession number**	**Gene name**	**Accession number**
spiC/ssaB	NP_460358	*sifB*	NP_460561	*fepG*	NP_752607
ssaC	NP_460359	*steB*	AAL20547	*fepD*	NP_752608
ssaD	NP_460360	*sseJ*	NP_460590	*entS*	NP_752609
ssaE	NP_460361	*steC*	NP_460656	*fepB*	NP_752610
sseA	NP_460362	*sopE2*	NP_460811	*entC*	NP_752611
sseB	NP_460363	*sspH2*	NP_461184	*entE*	NP_752612
sscA	NP_460364	*cheY*	YP_001006774	*entB*	NP_752613
sseC	NP_460365	*cheD*	YP_001006778	*entA*	NP_752614
sseD	NP_460366	*cheW*	YP_001006779	*slrP*	NP_459778
sseE	NP_460367	*cheA*	YP_001006780	*sopD2*	NP_459947
sscB	NP_460368	*fliA*	YP_001006726	*ompA*	AAF37887
sseF	NP_460369	*fliC*	YP_001006730	*pipB*	NP_460061
sseG	NP_460370	*fliC*	YP_001006728	*sopB/sigD*	NP_460064
ssaG	NP_460371	*fliG*	YP_001006742	*csgG*	NP_460110
ssaH	NP_460372	*fliM*	YP_001006748	*csgF*	NP_460111
ssaI	NP_460373	*fliN*	YP_001006749	*csgE*	NP_460112
ssaJ	NP_460374	*fliP*	YP_001006751	*csgD*	NP_460113
ssaK	NP_460376	*sopA*	NP_461011	*csgB*	NP_460114
ssaL	NP_460377	*manB*	YP_001007254	*csgA*	NP_460115
ssaM	NP_460378	*sseK2*	NP_461081	*csgC*	NP_460116
ssaV	NP_460379	*gmhA/lpcA*	NP_439337	*flgE*	YP_001006761
ssaN	NP_460380	*shdA*	NP_461448	*flgG*	YP_001006759
ssaO	NP_460381	*ratB*	NP_461449	*flgH*	YP_001006758
ssaP	NP_460382	*sinH*	NP_461452	*sifA*	NP_460194
ssaQ	NP_460383	*shdA*	NP_461448	*sipA/sspA*	NP_461803
ssaR	NP_460384	*gtrB*	NP_706258	*sipD*	NP_461804
ssaS	NP_460385	*sseK1*	NP_463026	*sipC/sspC*	NP_461805
ssaT	NP_460386	*manB*	YP_001007254	*sipB/sspB*	NP_461806
ssaU	NP_460387	*fepA*	NP_752600	*sicA*	NP_461807
steA	NP_460542	*fepC*	NP_752606	*spaS*	NP_461808

Table S3: Virulence genes found in the strain *Salmonella enterica* subsp. *enterica* serovar Rissen 19CS0416.

Virulence genes[3]			
**Gene name**	**Accession number**	**Gene name**	**Accession number**
spaR	NP_461809	*iroC*	NP_753167
spaQ	NP_461810	*iroN*	NP_753164
spaP	NP_461811	*pipB2*	NP_461706
spaO	NP_461812	*mig-14*	NP_461708
invJ	NP_461813	*avrA*	NP_461786
invI	NP_461814	*orgC*	NP_461789
invC	NP_461815	*orgB*	NP_461790
invB	NP_461816	*orgA*	NP_461791
invA	NP_461817	*prgK*	NP_461792
invE	NP_461818	*prgJ*	NP_461793
invG	NP_461819	*prgI*	NP_461794
invF	NP_461820	*prgH*	NP_461795
invH	NP_461821	*sptP*	NP_461799
sopD	NP_461866	*sicP*	NP_461800
cheD	YP_001006778	*gtrB*	NP_706258
mgtC	NP_462663	*fimI*	NP_459539
mgtB	NP_462662	*fimC*	NP_459540
misL	NP_462656	*fimD*	NP_459541
lpfA	NP_462541	*fimH*	NP_459542
lpfB	NP_462540	*fimF*	NP_459543
lpfC	NP_462539	*gtrB*	NP_706258
lpfD	NP_462538	*gtrA*	NP_706257
lpfE	NP_462537	*sseL*	NP_461229
iroB	NP_753168		

[3] The virulence factors were detected using Virulence Factors DB.

<table>
<tr><th colspan="2">SPI[4]</th><th colspan="3">Virulence genes[5]</th><th colspan="2">Mapping 19CS0416 against SPI-4[6]</th></tr>
<tr><th>SPI name</th><th>Accession number</th><th>SPI name</th><th>Gene</th><th>Accession number</th><th>Gene</th><th>Contig (Intervals bp)</th></tr>
<tr><td rowspan="3">SPI-2</td><td rowspan="3">gi|16763390:1461740-1501810</td><td rowspan="3">SPI-I</td><td rowspan="3">invA
sipC/sspC
sipB/sspB
orgA
sptP
sopE2</td><td rowspan="3">NP_461817
NP_461805
NP_461806
NP_461791
NP_461799
NP_460811</td><td>siiA</td><td>Contig 21 (15 -> 647)</td></tr>
<tr><td>siiB</td><td>Contig 21 (1,149 -> 2,033)
Contig 2 (1,149 -> 2,033)</td></tr>
<tr><td>siiC</td><td>Contig 2 (2,035 -> 3,355)</td></tr>
<tr><td>SPI-8</td><td>gi|16758993:3132530-3139414</td><td>SPI-3</td><td>mgtC
mgtB</td><td>NP_462663
NP_462662</td><td>siiD</td><td>Contig 2 (3,339 -> 4,616)</td></tr>
<tr><td rowspan="2">C63PI</td><td rowspan="2">AF128999</td><td rowspan="2">SPI-5</td><td rowspan="2">pipB
sopB/sigD</td><td rowspan="2">NP_460061
NP_460064</td><td>siiE</td><td>Contig 2 (4,633 -> 21,318)</td></tr>
<tr><td>siiF</td><td>Contig 2 (21,358 -> 23,424)</td></tr>
</table>

Table S4: SPIs were found in the strain *Salmonella enterica* subsp. *enterica* serovar Rissen 19CS0416.

[4] *Salmonella* pathogenicity islands were detected using SPIFinder.
[5]The virulence genes associated with pathogenicity island were detected using Virulence Factors DB.
[6] Geneious prime Version 11.0.3 was used to map 19CS0416 against SPI-4 (accession number KP234070). In the table are the detected genes with the corresponding contig number and interval in the sequence 19CS0416.

Gene Annotation[6]				
**Name**	**Minimum**	**Maximum**	**Length**	**Direction**
Shufflon-specific DNA recombinase CDS	75	1199	1125	forward
Hypothetical protein CDS	1211	1864	654	forward
putative nuclease CDS	1911	2426	516	forward
FIG00644392: hypothetical protein CDS	2521	3927	1407	forward
FIG00644392: hypothetical protein CDS	3967	4197	231	forward
FIG01045518: hypothetical protein CDS	4367	4942	576	forward
DNA topoisomerase III, TraE-type (EC 5.99.1.2) CDS	4947	7112	2166	forward
Haemolysin expression modulating protein CDS	7181	7387	207	forward
Hypothetical protein CDS	7807	8553	747	reverse
Lipid A phosphoethanolamine transferase, putative CDS	8601	10226	1626	reverse
Hypothetical protein CDS	10564	11004	441	forward
Hypothetical protein CDS	11047	15222	4176	reverse
Hypothetical protein CDS	15219	15560	342	reverse
Hypothetical protein CDS	15929	16330	402	forward
TraL CDS	16348	17100	753	forward
Hypothetical protein CDS	17102	17809	708	forward
Phage ea22 protein CDS	17829	18128	300	forward
Hypothetical protein CDS	18125	18604	480	forward
Phage protein CDS	18777	18965	189	forward
Phage protein CDS	18967	19254	288	forward
Phage ea22 protein CDS	19258	19578	321	forward
DNA-binding protein, CopG family CDS	19709	20146	438	reverse
Uncharacterized protein Ava 1937 CDS	20171	20353	183	reverse
Hypothetical protein CDS	20650	21105	456	forward
Hypothetical protein CDS	21304	21441	138	reverse
Hypothetical protein CDS	21518	21811	294	reverse

Table S5: Annotation of the contig_18 found in the strain *Salmonella enterica* subsp. *enterica* serovar Rissen 19CS0416.

Gene Annotation[6]				
**Name**	**Minimum**	**Maximum**	**Length**	**Direction**
Hypothetical protein CDS	21518	21811	294	reverse
Hypothetical protein CDS	21943	22056	114	reverse
FIG00640806: hypothetical protein CDS	22382	22624	243	reverse
FIG00765617: hypothetical protein CDS	22621	22875	255	reverse
FIG00643553: hypothetical protein CDS	22872	23414	543	reverse
Chaperone protein DnaJ CDS	23518	24033	516	reverse
Hypothetical protein CDS	24153	24266	114	forward
Hypothetical protein CDS	24437	24766	330	reverse
putative DnaJ-class molecular chaperone CDS	24898	25011	114	reverse
Chromosome partitioning protein ParA CDS	25355	26020	666	forward
unknown CDS	26094	26378	285	forward
Hypothetical protein CDS	26396	26968	573	reverse
Hypothetical protein CDS	27146	27361	216	reverse
FIG01048830: hypothetical protein CDS	27497	27658	162	reverse
Hypothetical protein CDS	28292	28615	324	forward
Replication initiation protein CDS	28713	29741	1029	reverse
Hypothetical protein CDS	29843	30175	333	forward
Hypothetical protein CDS	30873	31325	453	forward
Hypothetical protein CDS	31318	31533	216	forward
Hypothetical protein CDS	31526	31702	177	forward
Protein QmcA (possibly involved in integral membrane quality control) CDS	31705	32682	978	forward
Hypothetical protein CDS	32928	33053	126	forward
Hypothetical protein CDS	33056	33499	444	forward
Hypothetical protein CDS	33503	33673	171	forward
FIG00642148: hypothetical protein CDS	33684	34130	447	forward
Hypothetical protein CDS	34163	34420	258	forward

Gene Annotation[6]				
**Name**	**Minimum**	**Maximum**	**Length**	**Direction**
FIG00644780: hypothetical protein CDS	34700	34957	258	forward
Hypothetical protein CDS	34961	35956	996	forward
Hypothetical protein CDS	35962	36606	645	forward
Hypothetical protein CDS	36615	36839	225	forward
possible DNA primase CDS	37062	37871	810	reverse
IncN plasmid KikA protein CDS	37919	38218	300	forward
Hypothetical protein CDS	38221	39456	1236	forward
IncI1 plasmid conjugative transfer protein PilM CDS	39462	39899	438	forward
FIG00643686: hypothetical protein CDS	40018	40416	399	forward
Bores hole in peptidoglycan layer allowing typeIV secretion complex assembly (VirB1) CDS	40437	41021	585	forward
Major pilus subunit of type IV secretion complex (VirB2) CDS	41021	41311	291	forward
Hypothetical protein CDS	41382	41702	321	forward
ATPase required for both assembly of type IV secretion complex and secretion of T-DNA complex,VirB4 CDS	41708	44065	2358	forward
Hypothetical protein CDS	44097	44231	135	forward
Inner membrane protein forms channel for type IV secretion of T-DNA complex (VirB8) CDS	44231	44965	735	forward
Hypothetical protein CDS	44962	45732	771	forward
Inner membrane protein of type IV secretion of T-DNA complex, TonB-like, VirB10 CDS	45722	46861	1140	forward
ATPase required for both assembly of type IV secretion complex and secretion of T-DNA complex,VirB11 CDS	46880	47935	1056	forward
Coupling protein VirD4, ATPase required for T-DNA transfer CDS	47951	49909	1959	forward
Hypothetical protein CDS	49956	50492	537	forward
Hypothetical protein CDS	50485	52128	1644	forward
Hypothetical protein CDS	52167	53489	1323	forward
IncI1 plasmid pilus assembly protein PilP CDS	53473	53967	495	forward
Hypothetical protein CDS	53992	55530	1539	forward
Hypothetical protein CDS	55521	56630	1110	forward
IncI1 plasmid conjugative transfer prepilin PilS CDS	56675	57232	558	forward

Gene Annotation[6]				
**Name**	**Minimum**	**Maximum**	**Length**	**Direction**
IncI1 plasmid conjugative transfer putative membrane protein PilT CDS	57298	57780	483	forward
Hypothetical protein CDS	57784	58419	636	forward
IncI1 plasmid conjugative transfer pilus-tip adhesin protein PilV CDS	58432	59466	1035	forward

[6] Annotation was done by RAST Server
